# Ecotoxicity Assessment of Fe_3_O_4_ Magnetic Nanoparticle Exposure in Adult Zebrafish at an Environmental Pertinent Concentration by Behavioral and Biochemical Testing

**DOI:** 10.3390/nano9060873

**Published:** 2019-06-09

**Authors:** Nemi Malhotra, Jung-Ren Chen, Sreeja Sarasamma, Gilbert Audira, Petrus Siregar, Sung-Tzu Liang, Yu-Heng Lai, Geng-Ming Lin, Tzong-Rong Ger, Chung-Der Hsiao

**Affiliations:** 1 Department of Biomedical Engineering, Chung Yuan Christian University, Chung-Li 32023, Taiwan; nemi.malhotra@gmail.com; 2 Department of Biological Science & Technology, College of Medicine, I-Shou University, Kaohsiung 82445, Taiwan; jrchen@isu.edu.tw; 3 Department of Chemistry, Chung Yuan Christian University, Chung-Li 32023, Taiwan; sreejakarthik@hotmail.com (S.S.); gilbertaudira@yahoo.com (G.A.); 4 Department of Bioscience Technology, Chung Yuan Christian University, Chung-Li 32023, Taiwan; siregar.petrus27@gmail.com (P.S.); stliang3@gmail.com (S.-T.L.); 5 Department of Chemistry, Chinese Culture University, Taipei 11114, Taiwan; lyh21@ulive.pccu.edu.tw; 6 Laboratory of Marine Biology and Ecology, Third Institute of Oceanography, State Oceanic Administration, Xiamen 361005, China; 7 Center for Nanotechnology, Chung Yuan Christian University, Chung-Li 32023, Taiwan

**Keywords:** zebrafish, magnetic nanoparticles, behavioral analysis, ecotoxicity, neurotransmitters

## Abstract

Magnetic Nanoparticles (MNPs) are widely being investigated as novel promising multifunctional agents, specifically in the fields of development for theranostics, electronics, waste water treatment, cosmetics, and energy storage devices. Unique, superior, and indispensable properties of magnetization, heat transfer, and melting temperature make MNPs emerge in the field of therapeutics in future healthcare industries. However, MNPs ecotoxicity as well as behavioral toxicity is still unexplored. Ecotoxicity analysis may assist investigate MNPs uptake mechanism and its influence on bioavailability under a given set of environmental factors, which can be followed to investigate the biomagnification of MNPs in the environment and health risk possessed by them in an ecological food chain. In this study, we attempted to determine the behavioral changes in zebrafishes at low (1 ppm) or high (10 ppm) concentration levels of Fe_3_O_4_ MNPs. The synthesized Fe_3_O_4_ MNPs sized at 15 nm were characterized by the transmission electron microscope (TEM), the superconducting quantum interference device (SQUID) magnetometer, and the multiple behavior tests for novel tank, mirror biting, conspecific social interaction, shoaling, circadian rhythm, and short-term memory of zebrafish under MNPs chronic exposure were demonstrated. Low concentration MNP exposure did not trigger alteration for majority behavioral and biochemical tests in adult zebrafish. However, tight shoal groups were observed at a high concentration of MNPs exposure along with a modest reduction in fish exploratory behavior and a significant reduction in conspecific social interaction behavior. By using enzyme-linked immunosorbent assays (ELISA), we found a high dose of MNPs exposure significantly elevated cortisol, acetylcholine, and catalase levels while reducing serotonin, acetylcholine esterase, and dopamine levels in the brain. Our data demonstrates chronic MNPs exposure at an environmentally-relevant dose is relatively safe by supporting evidence from an array of behavioral and biochemical tests. This combinational approach using behavioral and biochemical tests would be helpful for understanding the MNPs association with anticipated colloids and particles effecting bioavailability and uptake into cells and organisms.

## 1. Introduction

Magnetic nanoparticles (MNPs) are approximately 1 to 100 nm in size with Fe, Co, Ni, and Al as the core with the crystal structure, physiochemical properties, and magnetic properties [1,2,3]. The iron core provides a contrast agent for imaging studies such as Magnetic Resonance Imaging (MRI) [4,5]. However, bare MNPs tend to form large aggregates in fluids, which impedes their utility [6]. Therefore, to avoid the aggregation of MNPs, a polymer (e.g. starch, dextran etc.) coating is applied over MNPs. The diffusion of surface-coated MNPs in fluids have been reported [3,7]. According to some studies, the toxicity of Fe_3_O_4_ MNPs is explained to be dependent on its surface coating and tissue examined, e.g., transcriptome sequence analysis to liver and gill transcriptomes of adult zebrafish on exposure to bare and starch-coated MNPs for seven days resulted in significant changes in a differentially expressed genes (DEGs) profile of both tissues [7]. On the other hand, some other studies have proposed developmental toxicity of nFe_2_O_3_ over zebrafish to be time dependent and dose dependent [8]. The regime of MNPs is gaining attention rapidly, but the ecotoxicity of MNPs is still under apprehension, including influence of environmental factors on bioavailability of MNPs and its uptake mechanism in the organism [9].

Currently, there is considerable interest in using MNPs for biomedical applications on drug delivery, bioimaging, and magnetic separation [10,11,12]. However, the potential toxicity of the MNPs, deleterious interactions within the body, with the environment, accumulation in organs, and excretion through the body (if any) are largely unknown. Aquatic animals, when exposed to environmental pollutants at primary junctures, respond rapidly. Therefore, they represent a suitable model to understand toxicity [13]. In a food chain, the released NPs might be transmitted in different organisms like algae, zooplankton, marine invertebrates, and fish [9,14,15]. Hence, the study of NPs ecotoxicity to the aquatic organism is of absolute importance [16]. The endocytosis mechanism for engulfment of MNPs might cause cell toxicity, which may lead to generation of reactive radicals [17] and interfere with normal functioning inside the body. To discern, how specialized magnetic nanoparticles interact with cells and the cell system within the body, it is essential to know the criteria of their safety with respect to environmental exposure. Usage of uncomplicated and well-controlled experimental conditions might lead to the considerate reasoning of potential effects of the environmental condition on aquatic organisms [18]. To understand the mechanism of toxicity in the aquatic organism, a set of comprehensive tests is required, which we have tried to achieve in the present study.

A small size (1 to 100 nm) of nanoparticles (NPs) and their physiochemical properties exhibit their beneficial traits in the category related to biomolecules such as nucleic acid and proteins positioned at the surface of the cell membrane, which might interfere with biological function of the cell causing disturbance in the normal functioning of the cell [19,20]. The probability of these interactions displays the potential to cause severe cell damage, depending on specific parameters used to design a nanoparticle [21,22]. Studies have reported the toxicity caused by MNPs with a different set of surface coatings. For example, dextran-coated Fe_3_O_4_ reduced cell proliferation and caused cell death analogous to that caused by uncoated iron oxide particles at 50 μg/mL [23]. In another study, lipid peroxidation, metal ion release, cell wall damage, and reactive oxygen species (ROS) production have been reported after MNPs exposure in vitro and in vivo [3,7,24,25,26,27]. Di-mercapto-succinic acid (DMSA)-coated Fe_3_O_4_ MNPs have been injected into the rat and it was subsequently accumulated in the spleen, liver, and lung tissues of the rat with no toxicity to the animals [27]. Recently, studies have evaluated the short-term toxicity of different engineered nanoparticles, including in chronic behavioral endpoint studies, genotoxicity, and biochemical studies [28,29].

In comparison to in vitro studies, in vivo studies provide robust and accurate results of toxicity parameters. Studies with model organisms, such as rat, mouse, and zebrafish are already in progress. Various factors such as exposure time, injection doses, environmental conditions, food, age, and stress levels affect the conclusions of results in these cases. Zebrafish possess excellent qualities of transparency at the embryonic stage, high fecundity (200 to 300 embryos in one hatching), cost-effectiveness, small size, and a rapid early developmental process [3,30,31]. The genome of zebrafish shares homology to that of the humans [7,32]. It has been stated that, gradually, zebrafish are becoming a more frequently used model organism to assess toxicology of nanomaterials [3,33]. In a previous study [8], the uncoated αFe_2_O_3_ MNPs were used to test acute toxicity in zebrafish embryos at different concentrations ranging from 0.1 to 100 mg/L with different exposure times, where the authors concluded increased mortality, delay in hatching, and malformation as developmental toxicities at concentrations ≥10 mg/L [8]. In another study done by Zhang et al., adult zebrafish were exposed to bare and starch-coated Fe_3_O_4_ MNPs for seven days. This was followed by cellular uptake, toxicity studies, and gene expression profiling [7]. Zhang et al. found that the bare and starch-coated NPs display different tissue toxicity and both types of NP can induce inflammation and oxidative stress [7]. However, up to now, no studies examined ecotoxicology of Fe_3_O_4_ MNPs with behavioral alterations in zebrafish so far.

In this study, we used adult zebrafish (*Danio rerio*) as a model organism to explore the potential ecotoxicity risk for MNPs by using a combinational approach with behavioral and biochemical tests. Fe_3_O_4_ MNPs were given at a low concentration of 1 ppm and a high concentration of 10 ppm to adult zebrafish for two weeks and, later, behavioral endpoints including novel tank exploration, mirror biting, predator avoidance, conspecific interaction, shoaling, the circadian rhythm, and short-term memory were measured. The neurotransmitters and other biomarkers in brain and other organs tissues were also measured for mechanistic exploration. To this end, evaluation of MNPs toxicity in adult zebrafish provides helpful information for specific modifications in healthcare and environmental techniques. These studies may help gather further information, which may help enhance efficient modification for commercial health care remedies and benefits.

## 2. Materials and Methods

### 2.1. Chemicals

Magnetic nanoparticles used in this study were synthesized in-house. Iron (III), Iron (II), and NaOH were purchased from Sigma Aldrich (St. Louis, MO, USA). Hydrazine (N_2_H_4_) was purchased from Alfa Aesar (Lancashire, UK). All the chemicals were used without any further modification. Double-distilled water was used with maintained heating and a regular N_2_ supply.

### 2.2. Synthetization and Characterization of Magnetic Nanoparticles

**Figure 1:**
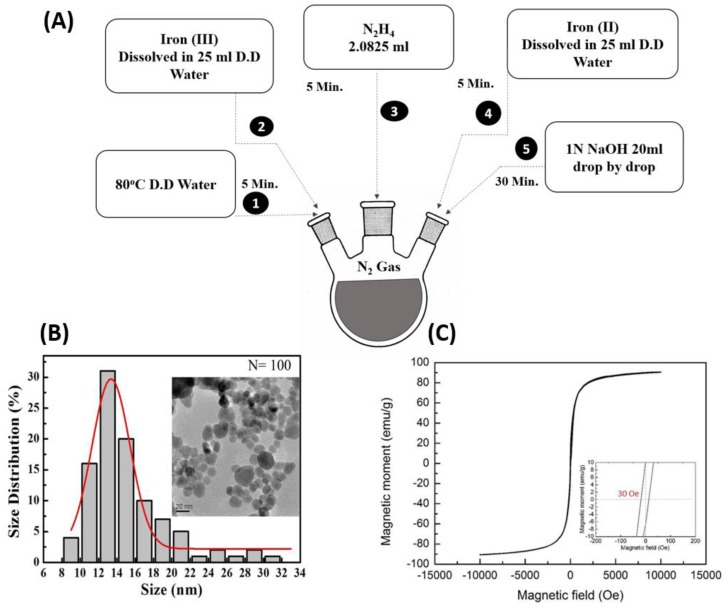
Physical property characterization of Fe_3_O_4_ magnetic nanoparticles (MNPs) used in this study. (**A**) Schematic diagram showing the protocol and process for MNPs synthesis. (**B**) Transmission electron microscopy (TEM) examination of the size distribution of the MNPs. Insert: showing the Fe3O4 MNPs particle size and shape under TEM (scale bar = 20 μm). (**C**) Hysteresis curve of the MNPs measured by superconducting quantum interference device (SQUID) magnetometry. Insert: showing the calculated magnetic field of the synthesized Fe3O4 MNPs.

A typical co-precipitation method was used for the synthesis of magnetic nanoparticles: 75 mL of double-distilled water was poured and maintained at 80 °C in a three-neck flask with a regular N_2_ supply and a magnetic stirrer. Iron (III) (FeCl_3_·6H_2_O, CAS No. 10025-77-1, purity 99%) was dissolved in 25 mL of double-distilled water and kept for five minutes. After five minutes, 2.0825 mL Hydrazine (N_2_H_4_, CAS No. 7803-57-8, purity 98+%) was added into the flask and incubated for five minutes. Subsequently, Iron (II) (FeCl_2_·4H_2_O, CAS No. 13478-10-9, purity 99%) was added in 25 mL double distilled water into the flask and kept for five minutes. Subsequently, 20 mL of 1N NaOH (CAS No. 1310-73-2, purity 99%) was added into the solution drop-by-drop with the help of a syringe pump for 30 min, to precipitate magnetic nanoparticles according to previous method [34]. This mixture was then allowed to cool before maintaining the pH using double distilled water. The desired pH value is 7, which needed to be confirmed at least three times from the mixture while adding double-distilled water. This mixture was then kept in a 50 molecular weight cut off (MWCO) bag for dialysis in distilled water overnight. The dialyzed MNPs solution was transferred into 50 mL test tubes and kept in the −80 °C freezer for a night. The frozen content was subsequently placed into the freeze dryer the following morning. After two days, we were able to recover the dried magnetic nanoparticles powder (procedure summarized in Figure 1A). The resultant powder form of MNPs were then characterized with transmission electron microscopy (TEM, H7100, Hitachi, Tokyo, Japan) to examine the shape, size, and dispersion of MNPs. In addition, Fourier-transform infrared spectroscopy (FTIR, IR-4200, Jasco, Easton, MD, USA) was performed to check the absorption and emission of MNPs on a broad-spectrum range. Afterward, a superconducting quantum interference device (SQUID, MPMS3, Quantum Design, Inc., San Diego, CA, USA) was used to confirm the successful magnetization of MNPs.

### 2.3. Zebrafish Ethics and Husbandry

The model organism Zebrafish was supervised and handled in accordance with the approved protocols and procedure by the Chung Yuan Christian University (Number: CYCU107030, issue date 24 December 2017). All the guidelines were followed, and procedures on animals were performed accordingly. Adult wild-type AB strain zebrafish (*Danio rerio*), aged around 6 to 7 months, were maintained in a recirculating aquatic system at 27.8 °C with a 10/14-h dark/light cycle, according to standards. Reverse osmosis (pH 7.0–7.5) was used to filter the circulating water in the aquarium. To ensure zebrafish take in MNPs, fish were fed fresh Artemia only once every two days.

### 2.4. Embryo Acute Toxicity Test

Breeding chambers were filled with fish water and was placed with a separator. Zebrafishes were separated based on their gender with 1 male (wild type, WT) and 2 female (WT) types. The container was then covered with a lid and kept secure. The following morning, the separator was removed and fishes were allowed to naturally mate. Fifteen minutes after the removal of the separator, we were able to collect the freshly laid eggs from the container. These embryos were first observed for any premature lethality. Afterward, these embryos were segregated in a group of 20, in six different Petri dishes. These different petri dishes were labelled as a control, 1, 10, 100, and 1000 ppm. The MNPs serial dilution was done with the help from a 1000 ppm stock solution. To determine the LC50, all these petri dishes were kept in an incubator for 96 h, with check point parameters of 24, 48, 72, and 96 h.

### 2.5. Zebrafish Exposure to Magnetic Nanoparticles

Healthy adult zebrafish were segregated in three different 50-L water tanks, containing 20 L of fish water each, in a group of 20 (animal density was less than 1 animal/L to reduce stress), prior to the addition of MNPs. To reduce the sacrifice of adult zebrafish by following the 3R (Replacement, Reduction, and Refinement) principles [35], we initially performed a zebrafish acute embryonic toxicity test for Fe_3_O_4_ MNPs, according to OECD236 guideline from 1 to 1000 ppm. The results show the 96-hour LC50 for Fe_3_O_4_ MNPs might be higher than 1000 ppm since there were no mortality detected even at the maximal dose tested (data not shown). According to the Taiwan environmental protection administration (EPA) regulation, the maximal concentration of iron in the industrial waste effluent should not exceed 10 ppm. Therefore, for adult zebrafish, we adapted Fe_3_O_4_ MNPs at 1 ppm as a low dose and 10 ppm as a high dose to perform the following behavioral and biochemical tests. The three different tanks were then labelled as control (0 ppm), low concentration MNPs (1 ppm), and high concentration MNPs (10 ppm). MNPs were weighed and diluted with double distilled water for sonication prior to their addition into the fish tanks. This fish water and dose of MNPs was changed every two days to avoid any chance of bacterial infection due to bad water quality from excess foods after feeding. After the successful completion of the experiment, a set of behavioral tests (novel tank test, shoaling test, predator avoidance test, social interaction test, aggressiveness test, circadian rhythm, and memory test) were scheduled and completed in all the three groups. Results were then compared between the control and treated groups.

### 2.6. Novel Tank Test

The novel tank test was conducted to observe the fish ability to acclimate to the novel environment. In this test, every fish was introduced into each test trapezoid tank: 22 cm along the bottom, 28 cm at the top, 15.2 cm high, and 15.9 cm along the diagonal side filled with ~1.25 L of fish water, as described in the previous method. The video recording was started immediately after tested fish were put into the test tank for one minute every 5 min until 30 min passed. Average speed, freezing time movement ratio, time in top duration, number of entries to the top, latency to enter the top, and total distance traveled in the top were analyzed later [36].

### 2.7. Aggressiveness Test

A mirror biting test was used to investigate the aggressiveness in the fish [37,38,39]. The same size tank from the previous test was filled with ~1.25 L of fish water and a mirror was placed at one side of the tank. Zebrafish were acclimated for one minute when introduced into the water tank. Afterward, zebrafish aggressive behavior was recorded for five minutes, according to our previous protocol and several important endpoints (average speed, mirror biting time percentage, longest duration in the mirror side, freezing time movement ratio, swimming time movement ratio, and rapid movement time ratio) were measured [36].

### 2.8. Predator Avoidance Test

Predator avoidance test was carried out to assess zebrafish reaction in response to both visual and olfactory cues when facing predators. In a similar manner to the aggressiveness test, the predator avoidance test was conducted in a versatile instrument with 10 same-size tanks from a previous test filled with ~1.25 L of fish water and segmented into two halves with a transparent separator [36]. A convict cichlid (*Amatitlania nigrofasciata*), which is the predator fish, was put into one side of the tank and tested zebrafish into the other. Both the predator and zebrafish were allowed to acclimatize initially for about one minute prior to the start of 5 min recording. Later, several important endpoints (average speed, predator approaching time percentage, average distance to the separator, a freezing time movement ratio, a swimming time movement ratio, and rapid movement time ratio) were measured.

### 2.9. Shoaling Test

Shoaling is an innate behavior for fish to swim together in order to reduce anxiety and the risk was captured by the predators. In order to observe the shoaling formation ability of zebrafish, this test was conducted. For this test, the same size tank from a previous test was filled with ~1.25 L of fish water. Afterward, fish in groups of three were introduced into each tank. After the initial one minute of acclimatization, the five-minute recording was started according to our previous protocol and several important endpoints (average speed, time in the top duration, average shoal area, average inter-fish distance, average nearest neighbor distance, and average furthest neighbor distance) were measured [36].

### 2.10. Social Interaction Test

A social interaction test was carried out in order to assess the zebrafish ability to interact with their conspecific. In the social interaction test, a glass separator as described in the predator avoidance test was used inside the same size tank from a previous test with ~1.25 L of fish water, according to our published protocol [36]. Tested fish was introduced in one side of the tank with a conspecific in another side. After they were acclimated for one minute, a 5-minute video recording was started. Afterward, several important endpoints (interaction time percentage, longest duration in the separator side, average speed, and average distance to the separator) were calculated.

### 2.11. Circadian Rhythm Locomotion Activity Test

To assess the sleep/awake behaviors of zebrafish after MNPs exposure, a circadian rhythm locomotion activity test was assessed on the 15th day of MNPs exposure. The test methodology was described in our previous published paper [39]. The dark/light cycle test apparatus consisted of six custom-made small fish tanks (20 × 10 × 5 cm), which were placed above a light box. For each tank, three fishes were tested. For the light cycle, a light emitting diode (LED) was used as a light source while an infrared light emitting diode (IR-LED) was used in the dark cycle. A 940-nm infrared camera with magnifying lens was located above the experimental setup to record the fish movements at 30 frames per second inside an incubator to maintain the temperature of the whole set-up. The one-minute videos were recorded every hour for 24 h. Afterward, several important endpoints (average speed, average angular velocity, meandering, freezing movement time ratio, swimming movement time ratio, and rapid movement time ratio) were measured in both light and dark cycles.

### 2.12. Short Term Memory Test

This test was carried out to assess zebrafish short-term memory after MNPs exposure. This test was performed by using passive avoidance setting, according to our previous publication [38]. Initially, 30 fish were randomly grouped into control and MNP exposed groups with 15 fish each. Afterward, fish in the experimental group were exposed to 10 ppm MNPs and were exposed to a shuttle box to perform a short-term memory test. The learning latency as well as the total number of electric shocks given for training and memory latency were recorded for a comparison between the control and 10 ppm MNPs exposed fish.

### 2.13. Tissue Preparation and Total Protein Determination

After the completion of all the behavioral analyses, zebrafish were sacrificed with an overdose concentration of 200 mg/L tricaine solution (A5040, Sigma, St. Louis, MO, USA). The whole brain was extracted for each independent assay. Three zebrafish whole brains were used to prepare a single homogenate for each sample, which were homogenized on ice in volumes of 50 (*v*/*w*) of phosphate-buffered saline (PBS) at a pH of 7.2 and bullet blender (Next Advance, Inc., Troy, NY, USA), with small magnetic beads. Samples were centrifuged at 4000 rpm for 20 min at 4 °C, and the supernatant was kept in microtubes in the freezer at −80 °C for further analysis. Total protein analysis was done using a Pierce BCA (bicinchonic acid) protein assay kit (23225, Thermo Fisher Scientific, Waltham, MA, USA). The color formation was analyzed at 562 nm using a microplate reader (Multiskan GO, Thermo Fisher Scientific), according to our previous method [38,39].

### 2.14. Quantification of Oxidative Stress Markers, Stress Hormone, and Ferric (Metal) Content in Brain Tissues

The reactive oxygen species (ROS) test was performed with ELISA kit (ZGB-E1561, Zgenebio Inc., Taipei, Taiwan), as per the manufacturer’s instructions. Stress hormones of cortisol, catecholamine, and metallothionine were measured by using commercial target-specific enzyme-linked immunosorbent assay (ELISA) kits (ZGB-E1562, ZGB-E1575, ZGB-E1562, Zgenebio Inc., Taipei, Taiwan). For hypoxia, energy, and DNA damage evaluation, hypoxia-inducible factor 1-alpha (HIF1-α), adenosine-5’-Triphosphate (ATP), creatine kinase (CK), and ssDNA contents were measured by using a commercial target-specific ELISA kit (ZGB-E1643, ZGB-E1580, ZGB-E1581, ZGB-E1595, Zgenebio Inc.). To detect the ferric (metal) content in the brain tissue sample, a colorimetric-based iron assay kit was used (A039-2, Nanjing Jiancheng Bioengineering Institute, Nanjing, China). The examination profile of catalase (CAT) and lipid peroxidation markers of thiobarbituric acid reactive substances (TBARS) was detected using a commercial target-specific ELISA kit (ZGB-E1582, Zgenebio Inc.). Catalase (CAT) analysis was done to check the presence of catalase protein in the tissue lysates of the brain tissue sample. TBARS determined the lipid peroxidation of respective brain tissue samples.

### 2.15. Determination of Neurotransmitters in Brain Tissues

The whole-brain tissue lysates were subjected to measure several different neurotransmitters’ activity by using ELISA kits in accordance with the instruction provided. A pool of brain tissue from three individual fish was considered as one sample. The tests were performed in triplicate using a total of nine fish per group to ensure consistency. Acetylcholinesterase (AChE), acetylcholine (ACh), dopamine (DA), serotonin (5-HT), GABA, and melatonin levels were determined by using an ELISA kit (ZGB-E1637, ZGB-E1585, ZGB-E1573, ZGB-E1572, ZGB-E1574, ZGB-E1597, purchased from Zgenebio Inc.), respectively, according to the specifications of the manufacturer. The absorbance was analyzed at 450 nm using a microplate reader (Multiskan GO, Thermo Fisher Scientific). Data was expressed in the defined calculative measurement units of total protein. All the assay kits used in our experiment are based on the sandwich ELISA method, which involves a specific antibody for the detection of the chemicals of interest. First, the target-specific antibodies were immobilized onto 96-well microplates. Afterward, the tissue homogenates (10 µL): Sample diluent (40 µL) and Horseradish peroxidase (HRP) (100 µL) conjugated target-specific antibodies were applied onto microplate and incubated at 37 °C for 1 h. After washing with washing buffer, chromogen A and B (50 µL) were applied onto the microplate and incubated at 37 °C for 15 min. Lastly, stop solution (50 µL) was applied to stop color development and the absorbance was analyzed at 450 nm using a microplate reader (Multiskan GO, Thermo Fisher Scientific). The relative concentration of the target protein was quantified by comparing the standard curve generated from the standard provided by the commercial kits.

### 2.16. Statistical Analysis

All statistical analyses were plotted and calculated by using the GraphPad prism (GraphPad Software version 7 Inc., La Jolla, CA, USA). Each fish group was individually compared to the control group, using either of one-way, two-way ANOVA or Kruskal-Wallis tests and continued with a follow-up test, which are Dunn’s or Dunnett’s multiple comparison test. A significant difference between control and treated groups was marked as * if *p* < 0.05, ** if *p* < 0.01, *** if *p* < 0.001, and **** if *p* < 0.0001.

## 3. Results

### 3.1. Characterization of Magnetic Nanoparticles (MNPs)

A co-precipitation method was used for the synthesis of Fe_3_O_4_ MNPs (Figure 1A). The MNPs obtained are spherical in shape with an average size of 15 ± 5 nm, as determined by Transmission Electron Microscopy (TEM) (Figure 1B). The picture in the inset of Figure 1B at the scale of 20 nm shows a good dispersion of MNPs after performing sonication. Next, with a superconducting quantum interference device (SQUID) magnetometer analysis, we obtained the hysteresis curve where applied magnetics field 30 Oe resulted in zero coercivity and remanence indicating no hysteresis in magnetization and good quality of the superparamagnetic MNPs (Figure 1C) [40].

### 3.2. MNPs Exposure Significantly Reduced the Novel Tank Exploration Behavior of Zebrafish

**Figure 2:**
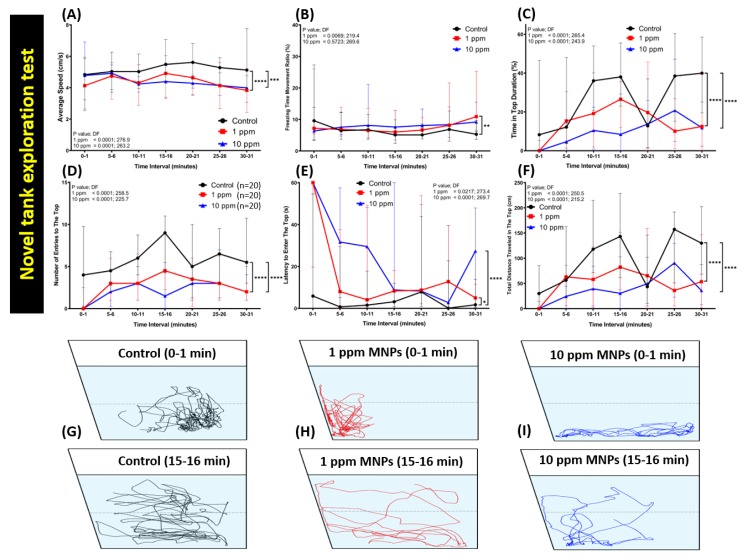
Behavior endpoint of control and Fe_3_O_4_ MNPs-exposed zebrafish in novel tank exploration test after two weeks’ exposure. (**A**) Average speed, (**B**) freezing time movement ratio, (**C**) time in top duration, (**D**) number of entries to the top, (**E**) latency to enter the top, and (**F**) total distance traveled in the top were analyzed. (**G–I**) The locomotion trajectories of control as well as 1 and 10 ppm MNPs-exposed fish in the novel tank test. The black line represents the control group, the red line represents the low concentration MNPs group (1 ppm), and the blue line represents the high concentration MNPs group (10 ppm). The data are expressed as the median with interquartile range and were analyzed by two-way ANOVA with Geisser-Greenhouse correction. To observe the main column (Fe_3_O_4_ MNPs) effect, Dunnett’s multiple comparison test was carried out. (*n* = 20, * *p* < 0.05, ** *p* < 0.01, *** *p* < 0.005, **** *p* < 0.001).

The novel tank test is a method to measure the exploration ability of fish after introduction to a new environment. Typically, when zebrafish are moved to a new environment, they display high anxiety and bottom dwelling behavior in the beginning, but, once they adapt to the new environment, they start to explore the tank and move toward the upper arena of the tank after their anxiety/stress is relieved [38,41,42]. After the specified period of two weeks, the MNPs exposed zebrafish were tested for their exploratory and anxiety behavior in the novel tank test. In comparison to the control group (0 ppm), the locomotion and exploratory activity of zebrafish with both concentrations were significantly reduced. However, there was a slight difference between low and high concentrations regarding a zebrafish behavior alteration result. In the low concentration group, a steep decrease in the average speed was observed (Figure 2A). This result was also supported with a significantly high level of a freezing time movement ratio exhibited by this group (Figure 2B), which was not displayed by a high concentration group. Meanwhile, exploratory behavior alteration was also shown by this group during the test, which was indicated by a significantly low level of time in the top duration, the number of entries to the top, and the total distance traveled in the top, and high level of latency to enter the top (Figure 2C–F). On the other hand, a high concentration group showed more robust alteration in their exploratory behavior than the low concentration group. Even though a significant decrement in time in the top duration, number of entries to the top, and total distance traveled in the top were also detected in this group (Figure 2C,D,F), a more severe effect regarding their latency to enter the top was exhibited by this group (Figure 2E). Furthermore, a low level of average speed and a high level of freezing time ratio displayed by this group also indicated that this group had locomotion activity alteration (Figure 2A,B). The locomotion trajectories for control, 1 ppm MNPs-exposed groups and 10 ppm MNP-exposed groups after 0 to 1 min (upper panel) and 15 to 16 min (bottom panel) acclimation were summarized in Figure 2G–I and Video S1. This result suggests the exposure to MNPs at either a low dose or a high dose elevates the anxiety level in zebrafish.

### 3.3. MNPs Exposure Did Not Alter Aggressiveness in Zebrafish

**Figure 3:**
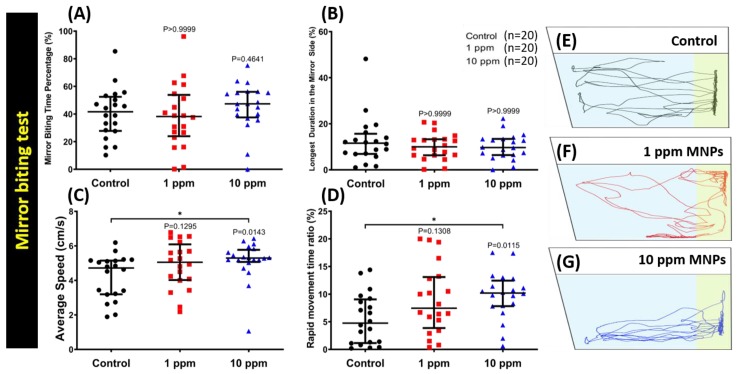
Mirror biting behavior endpoint comparisons between the control group, 1 ppm, and 10 ppm Fe_3_O_4_ MNPs-exposed zebrafish groups after two weeks of exposure. (**A**) Mirror biting time percentage, (**B**) longest duration in the mirror side, (**C**) average speed, and (**D**) the rapid time movement ratio were analyzed. The locomotion trajectories of control, 1, and 10 ppm MNPs-exposed fish in the mirror biting test were presented in (**E**–**G**), respectively. The data are expressed as the median with interquartile range and were analyzed by the Kruskal-Wallis test continued with Dunn’s multiple comparisons test as a follow-up test (*n* = 20, * *p* < 0.05).

The mirror biting assay is a simple and efficient method to test fish aggressiveness while monitoring the frequency of biting their own mirror images [38,42]. Unaltered mirror biting behaviors of the MNPs-exposed zebrafish was observed for both 1 and 10 ppm exposure (Figure 3). The average swimming speed was observed to be significantly faster in fish with high concentration MNPs (10 ppm) exposure compared to the control group (Figure 3C). In addition, the mirror biting time percentage (Figure 3A) and longest duration in the mirror side time (Figure 3B) are comparable to the case of the control group. The freezing time and swimming time duration (Figure A2A,B) were also observed to be almost the same in both groups. However, the rapid movement time (Figure 3D) showed a slight increase in the high concentration exposure group when compared to the control, which was consistent with their average speed result. The locomotion trajectories of control, 1 ppm, and 10 ppm MNPs exposed fish in the mirror biting test were summarized in Figure 3E–G and Video S2. This result suggests the MNPs exposure does not alter the mirror biting aggressiveness behavior in zebrafish, even though the swimming speed is faster in a high dose of the 10 ppm MNPs-exposed fish group.

### 3.4. MNPs Exposure Did Not Change the Predator Avoidance Behavior

**Figure 4:**
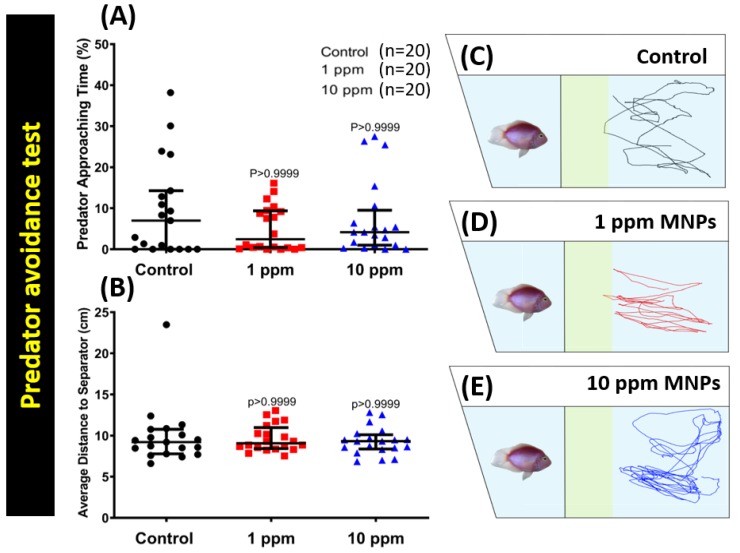
Predator avoidance behavior endpoint comparisons between control, 1 ppm, and 10 ppm Fe_3_O_4_ MNPs-exposed zebrafish groups after two weeks of exposure. (**A**) Predator approaching time percentage and (**B**) average distance to the separator were analyzed. The locomotion trajectories for the control, 1 ppm, and 10 ppm MNPs exposed fish in the predator avoidance test were presented in (**C**–**E**), respectively. The data are expressed as the median with interquartile range and were analyzed by the Kruskal-Wallis test with Dunn’s multiple comparisons test as a follow-up test (*n* = 19 for the control group, *n* = 20 for 1 ppm MNPs-exposed group, and *n* = 20 for 10 ppm MNPs exposed group).

Predator avoidance is an innate response for fish when facing their natural predator by showing high anxiety or even freezing behaviors. Similarly, zebrafish have an innate response such as freezing or anxiety when exposed to the sight of a natural predator [37,43]. This response is helpful in examining certain behavior alterations during the course of revelation to the predator. In this case, we exposed the different groups of zebrafish to the predator fish convict cichlid (*Amatitlania nigrofasciata*). Six independent measurements were analyzed during the predator avoidance test: average speed, predator approaching time percentage, average distance to the separator, freezing time movement ratio, swimming time movement, and the rapid movement time ratio. We found no exceptional behavior alteration in the predator avoidance test. No significant difference was detected in average speed (Figure A2C), predator approaching time (Figure 4A), average distance to the separator (Figure 4B), freezing time movement ratio (Figure A2D), swimming time movement ratio (Figure A2E), and rapid movement time ratio (Figure A2F) between the control and MNPs-exposed fish groups. The locomotion trajectories of control, 1 ppm, and 10 ppm MNPs exposed fish in the fear test were summarized in Figure 4C–E and Video S3. This result suggests the MNPs exposure does not change the predator avoidance behavior in zebrafish.

### 3.5. MNPs Exposure Tightened Shoaling Behavior in the High Concentration MNPs Group

**Figure 5:**
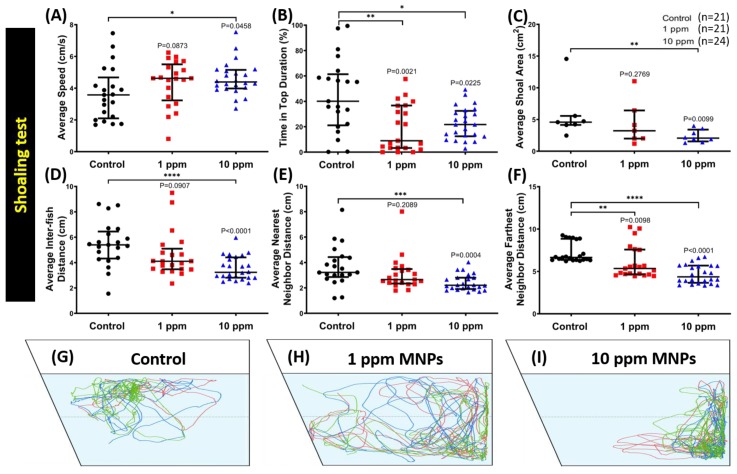
Shoaling behavior endpoint comparisons between the control group, 1 ppm, and 10 ppm Fe_3_O_4_ MNPs-exposed zebrafish groups after two weeks of exposure. (**A**) Average speed, (**B**) time in the top duration, (**C**) average shoal area, (**D**) average inter-fish distance, (**E**) average nearest neighbor distance, and (**F**) average furthest neighbor distance were analyzed. (**G–I**) The locomotion trajectories for the control, 1 ppm and 10 ppm MNPs exposed fish in the shoaling test. Groups of three fish were tested for shoaling behavior. The data are expressed as the median with interquartile range and were analyzed by the Kruskal-Wallis test, which continued with Dunn’s multiple comparisons test as a follow-up test (*n* = 21 for the control group and 1 ppm MNPs-exposed group, *n* = 24 for 10 ppm MNPs, * *p* < 0.05, ** *p*< 0.01, *** *p* < 0.001, and **** *p* < 0.0001).

Zebrafish is considered a highly social animal [44,45]. We tested the potential alteration of zebrafish social interaction after MNPs exposure with the shoaling test and conspecific social interaction test. Shoaling is an innate social behavior for fish to swim together in order to reduce anxiety and the risk of being captured by predators [46,47]. When zebrafish sense threats, they tend to swim together as a very tight group [37,48,49]. For the shoaling test, six endpoints were assessed in terms of average speed, time on top duration, average shoal area, average inter-fish distance, average nearest neighbor distance, and average farthest neighbor. Regarding their locomotion activity, a significant increase in average swimming speed in a high concentration-exposed group was observed when compared to the control group (Figure 5A). Furthermore, zebrafish spent less time at the top in both treatment groups (Figure 5B). In terms of shoaling formation, tighten shoals were formed by a high concentration group, which was indicated by a significant decrement in their average shoal area, average inter-fish distance, average nearest neighbor distance, and average farthest neighbor distance (Figure 5C–F). On the other hand, a low concentration of this particle only slightly affected their shoaling formation, which was shown by a low level of average nearest neighbor distance (Figure 5E). The locomotion trajectories for control, 1 ppm MNP-exposed, and 10 ppm MNP-exposed groups in the shoaling test were summarized in Figure 5G–I and Video S4. This result suggests MNPs exposure tighten the shoaling group behavior in zebrafish in a dose-dependent manner.

### 3.6. MNPs Exposure Reduced Conspecific Social Interaction Interest in Zebrafish

**Figure 6:**
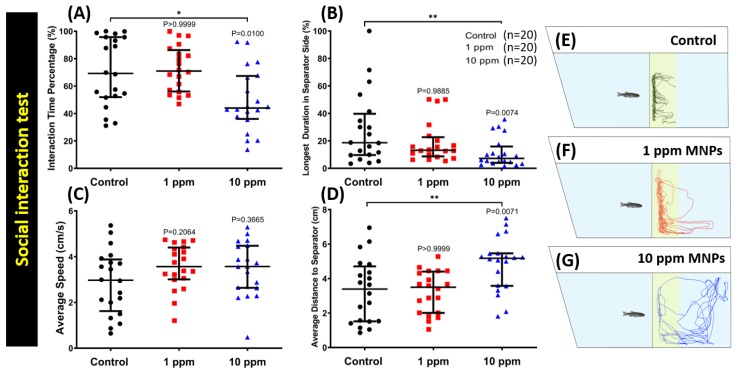
Behavior endpoint comparisons between the control group, 1 ppm, and 10 ppm Fe_3_O_4_ MNPs-exposed zebrafish groups after two weeks of exposure. (**A**) Interaction time percentage, (**B**) longest duration in the separator side, (**C**) average speed, and (**D**) average distance to the separator were analyzed. (**E–G**) The locomotion trajectories for the control, 1, and 10 ppm MNPs-exposed fish in the conspecific social interaction test. The data are expressed as the median with an interquartile range and were analyzed by the Kruskal-Wallis test, which continued with Dunn’s multiple comparisons test as a follow-up test (*n* = 20, * *p* < 0.05, ** *p* < 0.01).

Generally, zebrafish are highly social animals, even though they might display different behavior according to different habitats [37]. For the conspecific social interaction test, we introduced zebrafish into a specially designed tank with a transparent glass separator in the middle. The visiting frequencies of two isolated fish at each side were recorded and compared. According to our endpoint analysis with the MNPs exposed zebrafish groups (control, 1 ppm, and 10 ppm), considerable reduction in interaction time percentage (Figure 6A), longest duration in the separator side (Figure 6B), and increase in average distance to the separator (Figure 6D) were observed in the high concentration group while this phenomenon was not observed in the low concentration group. In addition, the average swimming speed (Figure 6C) of all the three groups remained essentially equal. The locomotion trajectories for the control, 1 ppm, and 10 ppm MNPs exposed fish in the conspecific social interaction test were presented in Figure 6E–G and Video S5. This result suggests MNPs exposure at a high dose can reduce conspecific social interaction interest in zebrafish.

### 3.7. MNPs Induced Dysregulation of Zebrafish Circadian Rhythm Locomotion Activity

**Figure 7:**
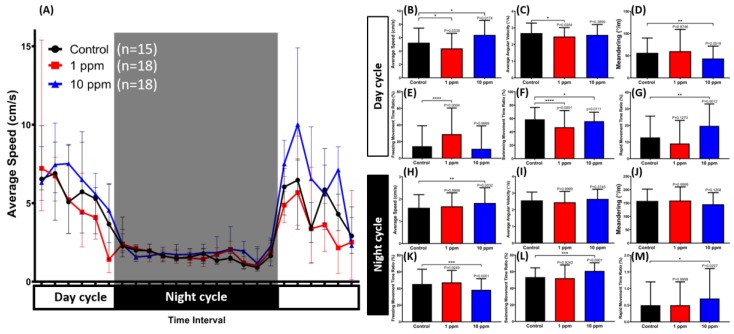
The circadian rhythm assay for Fe_3_O_4_ MNPs-exposed zebrafish after 14-day exposure. (**A**) Comparison of chronological changes of the average speed in light and dark cycles. The grey area shows the dark period while the white area shows the light period. Comparison of the (**B**) average speed, (**C**) average angular velocity, (**D**) meandering, (**E**) freezing movement time ratio, (**F**) swimming movement time ratio, and (**G**) rapid movement time ratio at the light cycle. Comparison of the (**H**) average speed, (**I**) average angular velocity, (**J**) meandering, (**K**) freezing movement time ratio, (**L**) swimming movement time ratio, and (**M**) rapid movement time ratio during the dark cycle. The data are expressed as the median with an interquartile range and were analyzed by a Kruskal-Wallis test, which continued with Dunn’s multiple comparisons test as a follow-up test (*n* = 15 for control, *n* = 18 for 1 and 10 ppm MNPs-exposed group, * *p* < 0.05, ** *p* < 0.01, *** *p* < 0.001, **** *p* < 0.0001).

Zebrafish is a typical diurnal fish species that display robust locomotion activity in the light cycle and sleep-like behavior in the dark cycle. For the circadian rhythm locomotion activity test, 12 behavior endpoints were identified, including average speed, average angular velocity, meandering, freezing movement time ratio, swimming movement time ratio, and rapid movement time ratio for both light and dark cycles. In agreement with our novel tank test result, a low concentration of MNPs exposure decreased zebrafish locomotion activity during the light cycle, which was indicated by a low level of average speed, average angular velocity, and swimming movement ratio as well as a high level of freezing movement time ratio (Figure 7B,C,E,F). On the other hand, hyperactivity behavior was displayed by the high concentration group during both light and dark cycles. In the light cycle, this behavior was shown by a significant increment of average speed and rapid movement ratio and a decrement of meandering and swimming movement ratio (Figure 7B,D,F,G). Meanwhile, a high level of average speed and rapid movement ratio displayed by this group during the dark cycle indicated that the fish did not display sleep-like behavior after being chronically subjected to a high concentration of MNPs, which was also supported with a low level of freezing time movement ratio and swimming movement ratio observed during the test (Figure 7H,K–M). However, there were no differences observed in both concentration groups regarding their average angular velocity and meandering in the dark cycle (Figure 7I,J). Taken together, a high concentration of MNPs exposure in zebrafish affected their circadian rhythm locomotion activity in both light and dark cycles while a low concentration was only affected in the light cycle (Figure 7A).

### 3.8. MNPs Reduced Short-Term Memory Retention in Zebrafish

**Figure 8:**
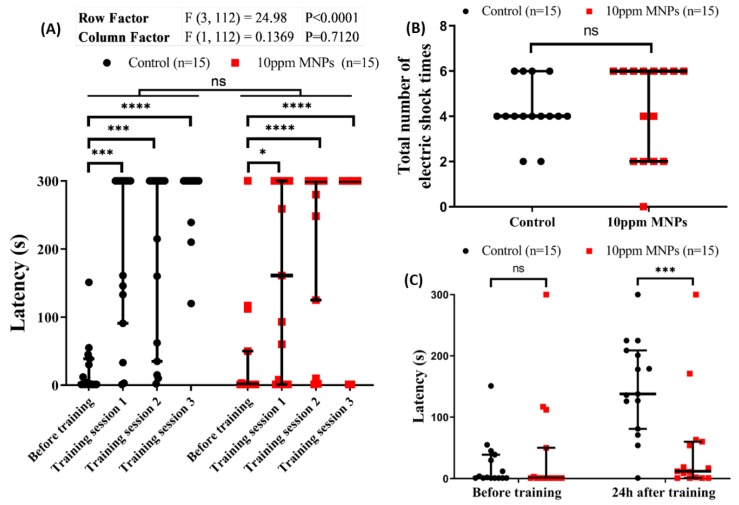
Passive avoidance test on zebrafish after 14 days of Fe_3_O_4_ MNPs exposure to evaluate short-term memory. (**A**) The latency of control (black) and MNPs-exposed (red) group in the dark chamber with electric shock punishment before and during training. (**B**) Total number of electric shocks given to zebrafish until passing the training session. (**C**) The latency of control (black) and MNPs-exposed (red) groups in the dark chamber with electric shock punishment before and one day after a training session. The data are expressed as the median with an interquartile range and significance were analyzed by a two-way ANOVA test (*n* = 15, * *p* < 0.05, *** *p* < 0.001, **** *p* < 0.0001).

In previous studies in humans or animals, long-term chronic exposure to heavy metal lead to memory loss and other neuropsychiatric problems [50,51,52]. In this case, we analyzed whether chronic MNPs exposure showed adverse effects on short-term memory in zebrafish. Results showed the chronic MNPs exposure at 10 ppm dose did not alter the learning process in terms of learning latency (Figure 8A) and total number of electric shocks given for training (Figure 8B). However, we found the memory latency in 10 ppm MNPs-exposed fish was significantly reduced when compared to the control group (Figure 8C). Therefore, we concluded that chronic MNPs exposure at a high dose of 10 ppm can induce dementia in zebrafish.

### 3.9. MNPs Exposure on Ferric (Metal) Content, Reactive Oxygen Species (ROS), and Stress Hormones in the Brain

**Figure A1:**
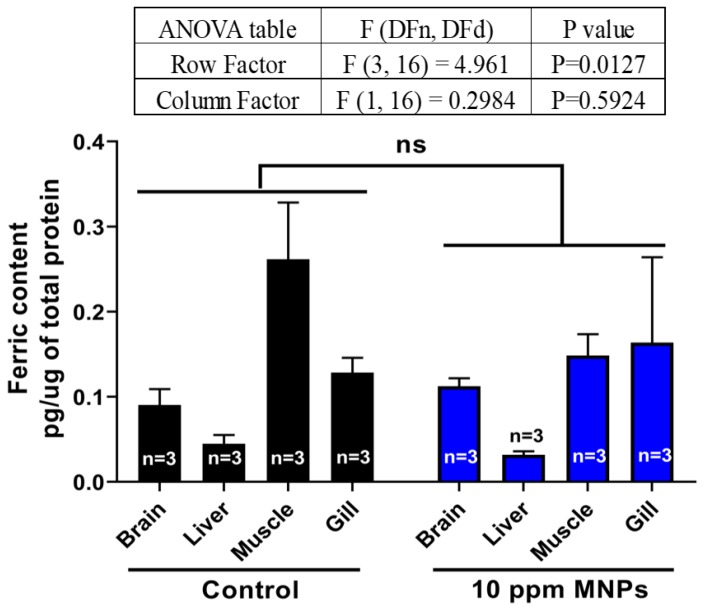
Comparison of ferric content among diverse tissues of brain, liver, muscle, and gill between the control and 10 ppm Fe_3_O_4_ MNPs exposed zebrafish. The ferric content in tissue was measured by the colorimetric method and normalized by total soluble protein content. The data are expressed as the mean ± S.E.M. and significance was tested by two-way ANOVA for multiple comparisons. The F factor and P value were also highlighted in the top panel to show the significance level. For each tissue, triplicates were given for ferric content measurement.

**Figure A2:**
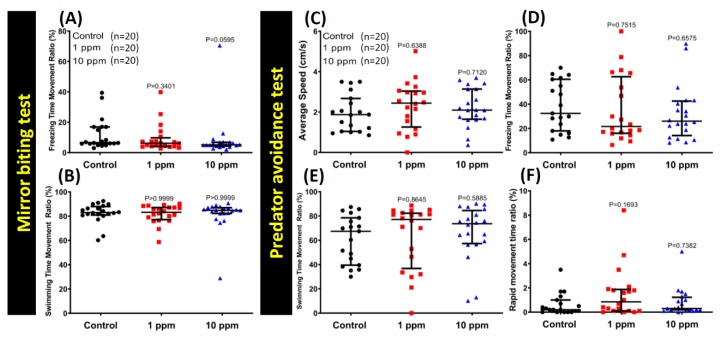
Mirror biting and predator avoidance tests behavior endpoint comparisons between control, 1 ppm, and 10 ppm Fe_3_O_4_ MNPs-exposed zebrafish groups after two weeks of exposure. (**A**) Freezing time movement ratio and (**B**) swimming time movement ratio was analyzed in the mirror biting test. (**C**) Average speed, (**D**) freezing time movement ratio, (**E**) swimming time movement ratio, and (**F**) rapid movement time ratio were analyzed in the predator avoidance test. The data are expressed as the median with interquartile range and were analyzed by the Kruskal-Wallis test with Dunn’s multiple comparisons test as a follow-up test (*n* = 20 for the all of the groups).

Zebrafish were sacrificed after completion of all the behavioral tests, and the ferric (metal) content in the brain tissue was quantified by using the ferric quantification colorimetric kit. However, no significant change of ferric content in the brain was detected in either a low (1 ppm) or a high (10 ppm) concentration of MNP-exposed fish (Table 1). Next, in addition to the brain, we also investigated the ferric contents in other tissue like liver, gill, and muscle to see the tissue-specific accumulation pattern for ferric ions (Figure A1). As a result, we found there was no significant difference for ferric content between the control and 10 ppm MNPs exposed fish by using the two-way ANOVA test, which demonstrates no entrapment of ferric ions in the tissues we surveyed. This hypothesis was supported by evidence from metallothionein quantification. Metallothionein is an inducible protein to chelate heavy metal to reduce its cellular toxicity. Enzyme Linked Immunosorbent Assay (ELISA) quantification results showed the metallothionein level displayed no significant difference between the control and MNPs-exposed fish (Table 1), which is consistent with the observation obtained from ferric ion quantification.

Moreover, we used ELISA to quantify the expression of biomarkers related to reactive oxygen species (ROS), anti-oxidative stress capacity, and stress response. ROS is an important factor for mediating metal-induced cellular responses, which lead to neoplasia or malignant tumors when it was accumulated over the threshold [53,54]. We found the relative ROS level (measured H_2_O_2_) in brain tissues of MNP-exposed fish, which did not display any significant difference in comparison to the control fish (Table 1). This result suggests MNPs exposure to zebrafish does not alter the ROS level in the brain. Next, we further analyzed catalase (CAT) expression, which has been reported to play a role in protecting cells from hydrogen peroxide toxicity, and is considered to be a marker of antioxidative response [55,56,57]. Significant increase of anti-oxidative enzyme of the CAT level in the high concentration MNPs (10ppm)-exposed group but not in the low dose MNPs (1ppm)-exposed group (one-way ANOVA F(2,6) = 5.91, *p* = 0.0380) (Table 1). In addition, we also measured the lipid peroxidation markers of thiobarbituric acid reactive substances (TBARS). TBARS is considered a good indicator of lipid peroxidation caused by oxidative stress in biological systems. TBARS showed no significant difference in all three groups of the ELISA test (Table 1).

Consequently, we analyzed the cortisol and catecholamine levels in fish brain to monitor the stress level in zebrafish after MNPs exposure. A significant increase in the cortisol hormone was observed in the high concentration (10 ppm) group when compared to the control and the low concentration group (1 ppm). In contrast, no significant difference was seen in catecholamine (CA) within the three groups. In addition, we also performed more tests for the tissue hypoxia marker of hypoxia-inducible factor 1-alpha (HIF-1α), tissue energy markers of adenosine-5′-triphosphate (ATP), and *creatine kinase* (CK), as well as the tissue damage marker of ssDNA. No significant difference between the control and MNPs exposed fish was detected in either HIF-1α, ATP, or CK, whereas ssDNA demonstrated a significant increase in the 10 ppm MNP-exposed group in comparison to 1 ppm and the control group, (*p* = 0.0429), which infers damage caused in DNA (Table 1). Taken together, our results suggested that MNPs exposure at a high dose (10 ppm) can induce stress responses in zebrafish to elevate cortisol levels in the brain. However, the majority of the markers related to free radical stress, lipid peroxidation, hypoxia, and energy balance remained unaltered in low dose groups.

### 3.10. MNPs Exposure on the Expression of Neurotransmitters in the Brain

Neurotransmitters affect a wide variety of both physical and psychological functions including heart rate, sleep, appetite, and behaviors such as mood and fear. To investigate MNPs exposure on neurotransmitters expression, the relative amount of neurotransmitter in the brain were measured biochemically using ELISA. The fixed amount of the total soluble protein in the brain was subjected to determine the expression level of acetylcholine esterase (AChE), acetylcholine (ACh), serotonin (5-HT), dopamine (DA), gamma-aminobutyric acid (GABA), and melatonin by ELISA.

AChE plays primary control in multiple physiological processes [58,59] and has been proven to impart swimming disorders in fish [59,60]. We observed a significant reduction in the amount of AChE in fish brain tissues at a high concentration of 10 ppm MNPs as well as at 1 ppm MNPs when compared to the control group (*p* = 0.03). Brain AChE decreased both in the low and the high concentration MNPs exposed groups, which indicates changes in the neuronal functions of brain tissue samples. Coinciding with the reduction of AChE, a significant elevation of ACh in the fish brain tissue was detected when a high dose of MNPs was given (* *p* = 0.01).

Serotonin (5-HT) showed a significant decline in high concentration (10 ppm) MNPs group in comparison to the control group (*p* = 0.02). A low level of 5-HT in zebrafish brain have been reported to display anxiety-like and depression-like syndromes [52,61]. The low level of serotonin in MNPs exposed fish lends support to the phenotype alterations showing longer bottom dwelling in a novel tank and reduced social interaction interest.

In comparison to the control group, a significant reduction of the dopamine level in the brain was detected in 10 ppm MNPs exposed group (*p* = 0.01). Dopamine is the major neurotransmitter in the brain for coping with stress [62]. The low level of dopamine after MNPs exposure might be related to an observed steep decrease in the exploratory behavior of zebrafish in the novel tank test and aggressiveness test. However, not much difference was observed in gamma-aminobutyric acid (GABA), which is an inhibitory neurotransmitter that is known for having a calming-effect on brain function. Melatonin plays a key role in regulating circadian rhythm and displays a high level in the night cycle and a low level in the day cycle [63,64,65]. ELISA data show that the melatonin levels display no significant difference after MNPs exposure. The dysregulation of the circadian rhythm behavior endpoints might be caused by some other reasons.

## 4. Discussion

In our study, we employed a panel of behavioral endpoint tests to observe the potential ecotoxicity of Fe_3_O_4_ MNPs in adult zebrafish for the first time. The test panel included: aggression test, predator avoidance test, shoaling test, novel tank test, social interaction test, and circadian rhythm and memory test. We then assessed the toxic effects on zebrafish when exposed to a low concentration (1 ppm) and a high concentration (10 ppm) for two weeks (14 days), and reported that the low concentration (1 ppm) MNPs dosage is very safe in the case of zebrafish. By contrast, a high concentration (10 ppm) revealed certain significant alterations in zebrafish neurological behaviors. The behavioral endpoint analyses in the shoaling test, novel tank test, and social interaction displayed a higher reduction and variation in the high concentration (10 ppm) group in comparison to the low concentration (1 ppm) and the control groups. A robust toxicity evaluation of MNPs-exposed zebrafishes has been lacking in previous studies. The findings in this study suggest that Fe_3_O_4_ MNPs showed significant alterations in the levels of brain neurotransmitters, especially when they are being given at a high dose (10 ppm), but are very safe at a low dose (1 ppm).

The toxicity of Fe_3_O_4_ MNPs is of concern because Ferric (iron) particles have been widely accepted as non-toxic. On a nano-scale, it is of great interest because MNPs portend a promising role in emerging healthcare trends. One study conducted by Kim et al. concluded that iron oxide particles of a 50-nm size do not exert toxicity on mice after intraperitoneal administration [66]. However, in other studies conducted by Zhu et al. showing iron oxide nanoparticles at 22 and 280 nm size induce significant toxicity in the model organism of rats after intratracheal instillation [67]. Some studies have also laid emphasis on the dosage of iron-oxide established in the model organism [68,69]. In addition, one zebrafish-related study conducted by Zhang et al. demonstrated that the Cmax Fe concentration in 4.0 mg/L treatment groups were slightly higher than that in 10.0 mg/L treatment groups, and that Fe concentration did not increase in the fish body with the rise in exposure concentration [29]. These intriguing findings suggest that zebrafish eliminate ingested ferric NPs. In another study, rats were injected with MNPs, and changes in iron level were evaluated in various tissues over a period of three weeks. The authors reported changes in iron level varied from tissue to tissue [70]. Moreover, no change in brain iron level during the initial first week of MNPs injection was reported, which suggests that intact MNPs do not cross the blood brain barrier. At later time points, a slow increase in iron levels was suggested, which may have occurred, due to binding of iron with transferrin (the main iron binding protein, which is actively involved in iron transport across body cells and tissues). The iron could then be transported across the blood brain barrier, since brain endothelial cells overexpress transferrin receptors [70,71]. MNPs are superparamagnetic in nature while free iron is expected to be paramagnetic. According to a study, saturation magnetization of the superparamagnetic component decreases with a decrease in MNP size. Larger sizes of MNPs are eliminated from the blood stream faster than smaller size MNPs [70,72].

Individual tissues in the body have different capacities to respond to oxidative stress [70]. To understand the cause of these behavioral changes, we performed biochemical assays for different neurotransmitters and stress hormone markers. The biochemical analysis showed an escalation in acetylcholine (ACh) and a decrease in acteylcholinesterase (AChE) activities, which suggests changes of nerve conduction ability, which can imply severe neurotoxicity and nervous system damage [59,73]. The thought-provoking results suggested an onset of dementia in the 10 ppm MNPs exposed group, which is antagonistically associated with the decrease of ACh and increase of AChE. Although several factors like a novel environment [74,75], age [76], dosage, or selective attentional functions [76,77] are associated with cholinergic neurotransmitter changes and might induce the measurement variations. However, in our study, we found the changes of cholinergic neurotransmitters and the dementia symptom observed in Fe_3_O_4_ MNPs-exposed zebrafish brain is consistent with previous findings collected from other metal pollutions [78,79,80]. This result supports cholinergic neurotransmitters that can be used as a good and reliable indicator to report the memory deficiency induced by Fe_3_O_4_ MNPs in zebrafish.

Dopamine deficiency has been reported to associate with multiple aspects of behavior such as loss of aggression, low locomotion speed, memory loss, sleeping problems, and reduced impulsive behaviors [62,81,82,83,84]. By ELISA, we found zebrafish display about two-folds reduction on the brain dopamine level. The dopamine deficiency is consistent with the behavioral phenotypes detected in a high dose of Fe_3_O_4_ MNPs exposed fish showing low locomotion activity (Figure 2), loss of aggression (Figure 3), circadian dysregulation (Figure 7), and loss of memory (Figure 8). 5-HT/Serotonin decrease in the high concentration 10 ppm group is also indicative of changes in neurological activities as seen in the less aggressive and social interaction behavior tests. Furthermore, these results are associated with depression. Taken together, all the evidence that supports zebrafish is an alternative vertebrate model to study Fe_3_O_4_ MNPs ecotoxicity at both biochemical and behavioral levels.

The findings in the behavioral test of aggressiveness, social interaction, and novel tank exploratory behavior of the zebrafishes can be related to the significant increase (around two-folds) in cortisol, which is an avidly recognized stress marker. However, the oxidative stress, lipid peroxidation, tissue hypoxia, and energy levels display unaltered processes even in a high-dose group. Based on the evidence collected from ferric content and metallothionine measurement, we showed Fe_3_O_4_ MNPs did not accumulated in the fish body (Figure 1A). We hypothesized that the long-term exposed fish already acclimated to Fe_3_O_4_ MNPs. This systematic compensation response might trigger some enzymes on fighting Fe_3_O_4_ MNPs pollution. For example, an elevation of anti-oxidative enzyme, catalase (CAT), was found in the brain of the high concentration group (10 ppm). Together, our results show that multiple factors involved in maintaining the stress, anxiety, and depression level in fish may be affected by exposure to MNPs. Further studies can be done to deepen our understanding of the molecular mechanism for Fe_3_O_4_ MNPs-induced toxicity by using the omic approach.

## 5. Conclusions

For the first time, we report the evaluation of chronic ecotoxicity of MNPs exposure on the brain of adult zebrafish. Herein, we have compiled a detailed synopsis of behavioral changes associated with neuronal functions upon exposure with two different concentrations of MNPs. No lethality was observed in either low or high concentration MNPs incubated zebrafish groups. Our results suggest that exposure of zebrafish to MNPs would not cause significant abnormalities at behavioral and biochemical levels in the low concentration 1 ppm group in comparison to the control group. However, significant changes in aggressiveness, speed, and locomotion behavior coupled with substantial changes in neurotransmitters and stress hormones have been induced in the zebrafish brains at a high concentration of 10 ppm. The observations suggested that MNP_S_ at environmental concentration exert minor effects on behavioral and biochemical activities of the fish brain. In the future, these observations might lead to some great insights for designing drug carriers for use in clinical settings. Nevertheless, our data highlight safety of Fe_3_O_4_ MNPs at fish neurobehavior levels that have not been investigated previously. Our studies have uncovered some minor behavioral alterations due to bare Fe_3_O_4_ MNPs exposure at high concentrations of 10 ppm, which leads us to require a further surface modification to increase its water solubility and biocompatibility to further reduce its biotoxicity. In addition, we proposed MNPs tracking in the fish body by using isotope labelling, which should be addressed in the future in order to better understand its dynamic distribution and clearance over time.

## Figures and Tables

**Table 1:** Various biomarker analytes in the brain tissue for adult zebrafish are exposed to low and high doses of Fe_3_O_4_ MNPs. The data were analyzed by one-way ANOVA and later subjected to post hoc significance analysis by using Dunnett’s multiple comparison test.

Biomarker	WT	MNP (1 ppm)	–	MNP (10 ppm)	–	Unit	Significance	ANOVA *F* (2,6) Value	*p* Value
Ferric ion	0.04 ± 0.01	0.09 ± 0.04	ns	0.04 ± 0.01	ns	pg/ug of total protein	NO	1.04	0.4107
Metallothionein	0.30 ± 0.04	0.32 ± 0.06	ns	0.27 ± 0.05	ns	pg/ug of total protein	NO	0.26	0.7737
ROS	86.19 ± 23.91	97.30 ± 16.03	ns	69.03 ± 18.82	ns	nmol/ug of total protein	NO	0.51	0.6220
Catalase	3.85 ± 0.46	5.17 ± 1.13	ns	9.35 ± 1.63	*	U/ug of total protein	YES	5.91	0.0380
TBARS	46.19 ± 15.77	41.76 ± 6.76	ns	34.15 ± 7.35	ns	ng/ug of total protein	NO	0.31	0.7383
ssDNA	2.06 ± 0.35	2.11 ± 0.24	ns	3.21 ± 0.20	*	U/ug of total protein	YES	5.56	0.042
ATP	240.70 ± 62.62	383.00 ± 108.50	ns	355.50 ± 84.78	ns	pg/ug of total protein	NO	0.74	0.5132
Creatine Kinase	10.30 ± 2.37	11.59 ± 2.13	ns	10.59 ± 3.10	ns	pg/ug of total protein	NO	0.06	0.9342
Hif-1α	34.20 ± 7.35	37.29 ± 6.06	ns	19.60 ± 0.10	ns	pg/ug of total protein	NO	2.94	0.1284
Cortisol	18.34 ± 1.30	19.42 ± 0.53	ns	37.15 ± 4.13	**	pg/ug of total protein	YES	17.42	0.0032
Catecholamine	10.33 ± 2.24	17.44 ± 2.73	ns	11.73 ± 1.39	ns	ng/ug of total protein	NO	2.94	0.1283
Acetylcholine esterase	43.35 ± 5.17	22.36 ± 5.84	*	18.95 ± 4.41	*	U/ug of total protein	YES	6.51	0.0313
Acetylcholine	19.60 ± 3.55	22.55 ± 0.70	ns	33.07 ± 1.86	*	U/ug of total protein	YES	9.06	0.0154
Dopamine	105.90 ± 14.10	97.79 ± 3.40	ns	55.26 ± 6.35	*	pg/ug of total protein	YES	8.84	0.0163
GABA	0.16 ± 0.05	0.16 ± 0.04	ns	0.08 ± 0.01	ns	U/ug of total protein	NO	1.08	0.3962
5-HT	1.39 ± 0.17	1.21 ± 0.03	ns	0.64 ± 0.17	*	ng/ug of total protein	YES	7.29	0.0247
Melatonin	36.32 ± 10.99	33.61 ± 4.73	ns	34.20 ± 5.60	ns	pg/ug of total protein	NO	0.03	0.9660

ns: no significance. The data are expressed as the mean ± S.E.M. and significance were analyzed by a one-way ANOVA test. * *p* < 0.05, ** *p* < 0.01.

## Supplementary Materials

The following are available online at https://www.mdpi.com/2079-4991/9/6/873/s1. Video for the novel tank exploratory behavior can be found in Video S1. Video for the aggressiveness in the mirror biting test can be found in Video S2. Video for the fear behavior in the predator avoidance test can be found in Video S3. Video for the conspecific social interaction can be found in Video S4. Video for the shoaling behavior can be found in Video S5.
